# Alternative splicing events expand molecular diversity of camel CSN1S2 increasing its ability to generate potentially bioactive peptides

**DOI:** 10.1038/s41598-019-41649-5

**Published:** 2019-03-27

**Authors:** Alma Ryskaliyeva, Céline Henry, Guy Miranda, Bernard Faye, Gaukhar Konuspayeva, Patrice Martin

**Affiliations:** 1 INRA, UMR GABI, AgroParisTech, Université Paris-Saclay, 78350 Jouy-en-Josas, France; 2 INRA, MICALIS Institute, Plateforme d’Analyse Protéomique Paris Sud-Ouest (PAPPSO), Université Paris-Saclay, 78350 Jouy-en-Josas, France; 3 CIRAD, UMR SELMET, 34398 Montpellier Cedex 5, France; 4 Al-Farabi Kazakh National University, Biotechnology department, 050040 Almaty, Kazakhstan

## Abstract

In a previous study on camel milk from Kazakhstan, we reported the occurrence of two unknown proteins (UP1 and UP2) with different levels of phosphorylation. Here we show that UP1 and UP2 are isoforms of camel α_s2_-CN (α_s2_-CNsv1 and α_s2_-CNsv2, respectively) arising from alternative splicing events. First described as a 178 amino-acids long protein carrying eight phosphate groups, the major camel α_s2_-CN isoform (called here α_s2_-CN) has a molecular mass of 21,906 Da. α_s2_-CNsv1, a rather frequent (35%) isoform displaying a higher molecular mass (+1,033 Da), is present at four phosphorylation levels (8P to 11P). Using cDNA-sequencing, α_s2_-CNsv1 was shown to be a variant arising from the splicing-in of an in-frame 27-nucleotide sequence encoding the nonapeptide ENSKKTVDM, for which the presence at the genome level was confirmed. α_s2_-CNsv2, which appeared to be present at 8P to 12P, was shown to include an additional decapeptide (VKAYQIIPNL) revealed by LC-MS/MS, encoded by a 3′-extension of exon 16. Since milk proteins represent a reservoir of biologically active peptides, the molecular diversity generated by differential splicing might increase its content. To evaluate this possibility, we searched for bioactive peptides encrypted in the different camel α_s2_-CN isoforms, using an *in silico* approach. Several peptides, putatively released from the C-terminal part of camel α_s2_-CN isoforms after *in silico* digestion by proteases from the digestive tract, were predicted to display anti-bacterial and antihypertensive activities.

## Introduction

Recently, combining different proteomic approaches, the complexity of camel milk proteins was resolved to provide a detailed characterization of fifty protein molecules belonging to the 9 main milk protein families, including caseins: κ-, α_s2_-, α_s1_- and β-CN and two unknown proteins (UP1 and UP2), exhibiting molecular masses around 23,000 Da^1^. Since UP1 and UP2 co-eluted in RP-HPLC with α_s_-CN and displayed different phosphorylation levels, it was tempting to consider that these proteins could originate in CN. However, based on their molecular weight, UP1 and UP2 could be larger isoforms of α_s2_-CN or smaller isoforms of α_s1_-CN.

**Figure 1:**
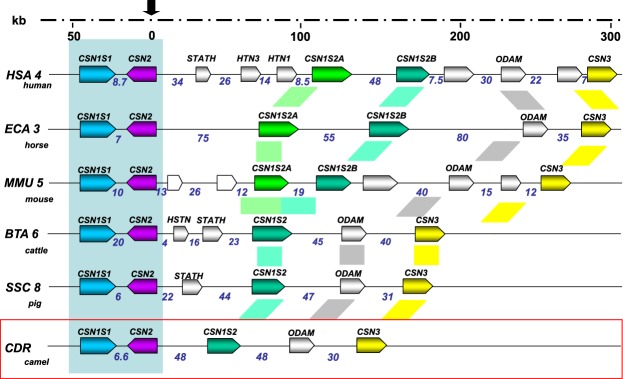
Evolution of the casein locus organization. Casein locus organization of human (*Homo sapiens*), horse (*Equus caballus*), mouse (*Mus musculus*), cattle (*Bos taurus*), pig (*Sus scrofa*) and camel (*Camelus dromedarius*) genomes (adapted from Martin, Cebo and Miranda^7^ and Lefèvre *et al*.^50^ with additional genomic information from the NCBI) was compared. Genes are given as colored arrow boxes, showing the orientation of transcription. Putative genes based on similarity are indicated as empty boxes. Intergenic region sizes are given in kb.

However, the hypothesis of an additional casein in camel milk encoded by a supplementary gene could not be ruled out. Indeed, genes encoding CN are tightly linked on the same chromosome, BTA6 in cattle, CHI6 in goats^2,3^ and HSA4 in humans^4^. The evolution of the CN gene cluster (Fig. 1) is postulated to have occurred by a combination of successive intra- and inter-genic exon duplications^5–7^. In some mammals, including horses, donkeys, rodents and rabbits, there are two α_s2_-CN encoding genes differentiating in size (*CSN1S2*-like or *CSN1S2A* and *CSN1S2B*), which may have arisen by a gene-duplication event that has occurred prior to the split of Eutherian mammalian species^5^. The second *CSN1S2*-like gene was lost in the Artiodactyla, including the camel, while further divergence occurred in both copies in the other species. In humans, there are also two *CSN1S2* genes albeit no evidence of protein expression exists^6^.

Alternative splicing is a process by which multiple mRNA isoforms are generated. It is a powerful means to extend protein diversity. Such a process which is another possibility to increase the number of molecular species has been frequently reported to occur, as far as caseins are concerned, especially α_s_-CN^8–10^, without really knowing whether it is a fortuitous or a scheduled event to expand molecular diversity and functionality of milk proteins. To substantiate the hypothesis according to which UP1 and UP2 might originate in CN and more precisely in α_s_-CN, we undertook characterizing more precisely these proteins.

In addition to their nutritional value, an increasing number of therapeutic effects and a variety of potential activities^11,12^ are attributed to milk proteins as well as to milk-derived bioactive peptides encrypted in milk protein sequences^13^. Caseins, and especially α_s_-CN, have been shown to be a reservoir of bioactive peptides^13–15^, it is therefore legitimate to wonder whether these so far unknown and putatively derived α_s_-CN sequences could be responsible for the occurrence of novel bioactive peptides accounting for the original properties of camel milk. Recent studies have indeed shown that healing properties assigned to camel milk, which is consumed fresh or fermented and traditionally used for the treatment of tuberculosis, gastroenteritis, and allergies, in many countries, are proved^16^. Whereas there is a substantial literature on bioactive peptides derived from bovine milk proteins^13^ and more or less comprehensive databases of milk bioactive peptides exist^17–20^, studies aiming at identifying peptides derived from camel milk proteins having potential health-promoting activities are scarce. Investigations mainly focused on caseins (α_s1_-, β- and κ-CN), and data available to date mostly concern *in vitro* and *in silico* antioxidant, antihypertensive and antimicrobial activities^16,21^. Therefore, using an *in silico* approach, we searched for potential biological activities of sequences generated from alternative splicing of primary transcript encoding α_s_-CN.

## Results and Discussion

### What gene(s) do UP1 and UP2 arise from

**Figure 2:**
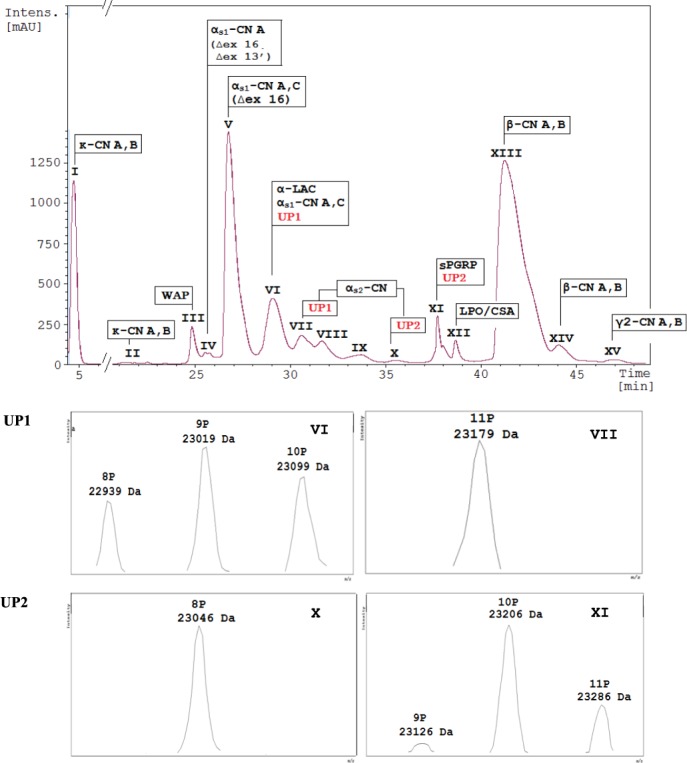
LC-ESI-MS profile of dromedary milk proteins. The chromatogram displays the presence of 15 major milk protein fractions labeled from I to XV, with retention times from 4.50 to 48.71 min, respectively. Deconvolution of multicharged ions spectra with emphasis on phosphorylation degrees (P) of two unknown proteins (UP1 and UP2) which are related to chromatographic peaks VI and VII, X and XI respectively.

The mass accuracy has allowed distinguishing about fifty protein molecules corresponding to isoforms of 9 protein families (κ-CN, WAP, α_s1_-CN, α-LAC, α_s2_-CN, PGRP, LPO/CSA, β-CN and γ2-CN) from LC-MS analysis as shown in Fig. 2. The presence of two unknown proteins UP1 and UP2 with different phosphorylation levels was reported in our previous study^8^. Regarding UP1, molecular masses ranged between 22,939 and 23,179 Da, whereas UP2 masses ranged between 23,046 Da and 23,366 Da (Table 1), with successive increments of 80 Da (mass of one phosphate group). The eluting range of these two proteins was between 28.53–37.16 min, within the elution times of α_s1_- and α_s2_-CN, which confirms our first hypothesis about their α_s_-CN origin. However, UP1 and UP2 masses exceeded the observed mass of the major isoform of α_s2_-CN with 8P (21,906 Da) by 1,033 Da and 1,300 Da, respectively, and were lighter than the C variant of α_s1_-CN-6P (25,773 Da) by 2,834 Da and 2,567 Da, respectively^1^. Even though it was not possible to exclude a splicing event leading to the inclusion of an additional exon sequence in the α_s2_-CN mRNA, the most probable hypothesis was the occurrence of exon-skipping event(s) affecting α_s1_-CN mRNA and, leading to the loss of a peptide sequence accounting for a reduction of at least 2,567 Da. A possible scenario was the skipping of exon 3 on the short isoform of α_s1_-CN C already impacted by a cryptic splice site usage (∆CAG encoding Q83). The molecular mass of the protein proceeding from such a messenger (23,205 Da) corresponded to the mass of UP2 + 160 Da (23,206 Da). However, sequencing cDNA encoding α_s1_-CN isoforms failed to reveal the existence of a messenger in which exon 3 was lacking. Therefore, the alternative possibility, in other words the α_s2_-CN avenue, had to be explored.

**Table 1:** Analysis of molecular masses contained in peaks V–XI of dromedary milk sample from the Shymkent region.

Peak	Ret.Time, min	Observed M_*r*_, Da	Theoretical M_*r*_, Da	Protein description	UniProt accession	Intensity
V	26,31	24,547	24,547	α_s1_-CN C -short isoform (∆ex 16), 5P, splice variant (∆Q83)		3,954
		24,561	24,561	α_s1_-CN A - short isoform (∆ex 16), 5P, splice variant (∆Q83)		4,385
		24,627	24,627	α_s1_-CN C - short isoform (∆ex 16), 6P, splice variant (∆Q83)		16,348
		24,640	24,641	α_s1_-CN A - short isoform (∆ex 16), 6P, splice variant (∆Q83)		17,422
		24,675	24,675	α_s1_-CN C - short isoform (∆ex 16), 5P		7,758
		24,689	24,689	α_s1_-CN A - short isoform (∆ex 16), 5P		8,004
		24,722	24,721	α_s1_-CN A - short isoform (∆ex 16), 7P, splice variant (∆Q83)		4,453
		**24,755**	**24,755**	**α**_**s1**_**-CN C - short isoform (∆ex 16), 6**P	**K7DXB9**	**34,653**
		**24,768**	**24,769**	**α**_**s1**_**-CN A - short isoform (∆ex 16), 6**P	**O97943-2**	**37,452**
		24,835	24,835	α_s1_-CN C - short isoform (∆ex 16), 7P		5,026
		24,849	24,849	α_s1_-CN A - short isoform (∆ex 16), 7P		4,851
VI	28.80	**14,430**	**14,430**	**α-LAC**	**P00710**	**12,948**
		**22,939**	n/a*	**UP1**	**n/a**	**2,676**
		23,019	n/a	UP1, +80 Da		2,408
		23,099	n/a	UP1, +160 Da		958
		25,645	25,645	α_s1_-CN C, 6P, splice variant (∆Q83)		1,736
		25,659	25,659	α_s1_-CN A, 6P, splice variant (∆Q83)		1,057
		25,693	25,693	α_s1_-CN C, 5P		916
		**25,772**	**25,773**	**α**_**s1**_**-CN C, 6**P		**5,014**
		25,787	25,787	α_s1_-CN A, 6P	O97943-1	1,509
VII	30.07	21,826	21,825	α_s2_-CN, 7P		709
		**21,906**	**21,905**	**α**_**s2**_**-CN, 8**P	**O97944**	**4,222**
		21,985	21,986	α_s2_-CN, 9P		289
		23,179	n/a	UP1, +240 Da		1,430
VIII	31.26	21,986	21,985	α_s2_-CN, 9P	O97944	866
		22,066	22,065	α_s2_-CN, 10P		3,682
IX	33.04	22,066	22,065	α_s2_-CN, 10P		120
		22,146	22,145	α_s2_-CN, 11P		1,408
X	34.85	22,226	22,225	α_s2_-CN, 12P		806
		23,046	n/a	UP2	n/a	295
XI	37.15	**19,143**	**19,143**	**PGRP**	**Q9GK12**	**3,659**
		23,126	n/a	UP2, +80 Da		150
		**23,206**	**n/a**	**UP2**, +**160 Da**		**1,162**
		23,286	n/a	UP2, +240 Da		940

*n/a - not applicable*.

### UP1 and UP2: new camel α_s2_-CN splicing variants

**Figure 3:**
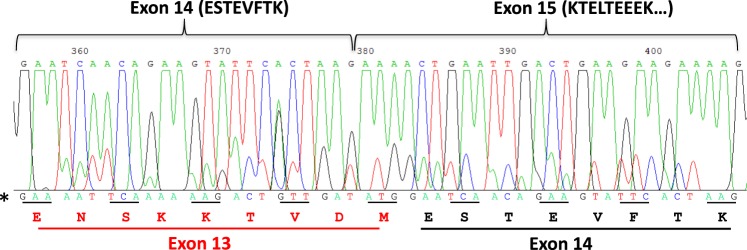
Sequence of *C*. *dromedarius* α_s2_-CN cDNA spanning exons 14 and 15 (main sequence). A secondary sequence (*) identified by manual reading of overlapping weak signals is given below the main sequence, showing the existence of transcripts, in which exon 13 is included. The corresponding aa sequence is given below. cDNA sequences encoding CSN1S2sv1 were submitted to NCBI Genbank with the following submission IDs: BankIt2160486 Seq. 1 MK077758 (*C*. *bactrianus*) and BankIt2160533 Seq. 1 MK077759 (*C*. *dromedarius*).

**Figure 4:**
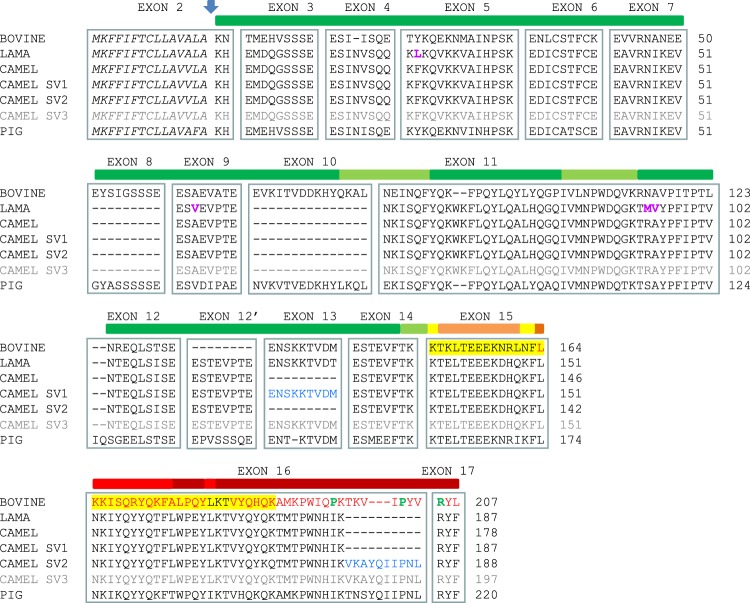
Multiple alignment of α_s2_-CN protein sequences from different Artiodactyls species. *Bos taurus* (M16644), *C*. *dromedarius* (O97944 and splicing variants identified in the present study), *Lama glama* (A0A0D6DR01), and *Sus scrofa* (X54975) protein sequences are compared. Camel α_s2_-CN putative isoform (α_s2_-CNsv3) comprising both additional sequences, encoded by exon 13 and exon16 extension, is in grey. Sequences are split into blocks of amino acid residues to visualize the exon modular structure of the protein as deduced from known splice junctions of the bovine gene^51^. Exon numbering (top of blocks) is that of the bovine gene taken as reference for Artiodactyls. Amino-acid sequences characterizing UP1 (α_s2_-CNsv1) and UP2 (α_s2_-CNsv2) encoded by exon 13 and the extension of exon 16, respectively, are given in blue. Italics indicate the signal peptides, for which the vertical blue arrow points out the cleavage site. Dashes indicate missing aa residues. Amino acid mutations distinguishing camel and lama α_s2_-CN are in fuchsia. The highest sequence antimicrobial peptide density is indicated by red on a heat map above the bovine protein sequence. The regions of Bioactive peptides encrypted in bovine α_s2_-CN f(150–188) with antibacterial activities reported by Zucht *et al*.^39^ are highlighted in yellow, while two antibacterial domains f(164–179) and f(183–207) described by Recio and Visser^40^ are indicated in red. Amino acid residues increasing significantly antibacterial potency are in green. Full-length mature CSN1S2sv1 and CSN1S2sv2 aa sequences were submitted to Expasy UniProtKB database as splicing variants of *C*. *dromedarius* CSN1S2 with the following submission IDs: SPIN200013828 and SPIN200013835, respectively.

Amplification of camel α_s2_-CN cDNA revealed the presence of a major PCR fragment (*ca*. 620 bp) and several minor PCR products differing in size between *ca*. 670 bp and 710 bp (Supplementary Data S1). Sequencing of PCR fragments generated two different nucleotide sequences: first identical from the forward primer to nucleotide 359, and then overlapping and shifted by 27 nucleotides (Fig. 3). The main sequence corresponded to the 193-aa α_s2_-CN (including the signal peptide) reported by Kappeler *et al*.^22^. The second sequence, with weaker signals, showed the insertion of the following sequence: GAA AAT TCA AAA AAG ACT GTT GAT ATG, between exons 12′ and 14. Thus, this insertion introduced an additional peptide sequence (ENSKKTVDM), identical to the aa sequence encoded by exon 13 in the bovine *CSN1S2* gene (Fig. 4). The level of exon 13 conservation in both species appeared to be extremely high. This exon is also present in the predicted sequence of the *CSN1S2* gene from the *Camelus ferus* genome (NCBI Reference Sequence: XP_014418048.1) and the lama gene transcript (GenBank: LK999989.1) with two point mutations. The first mutation concerning the fourth codon (AAA = >AAT) is silent and the second one, that is a missense mutation, regards the last codon (ACG = >ATG), leading to T = >M substitution^23^. Exon 13 is present in one of the two copies of the *CSN1S2* gene of most mammalian species. In mice, rats and rabbits the aa sequence encoded by this exon is present in CSN1S2*-*like (or CSN1S2A) protein but not in CSN1S2B^24^. The insertion of this sequence leads to the increasing of the molecular mass of α_s2_-CN by 1,033 Da, exactly the mass difference observed between α_s2_-CN-8P and UP1.

**Figure 5:**
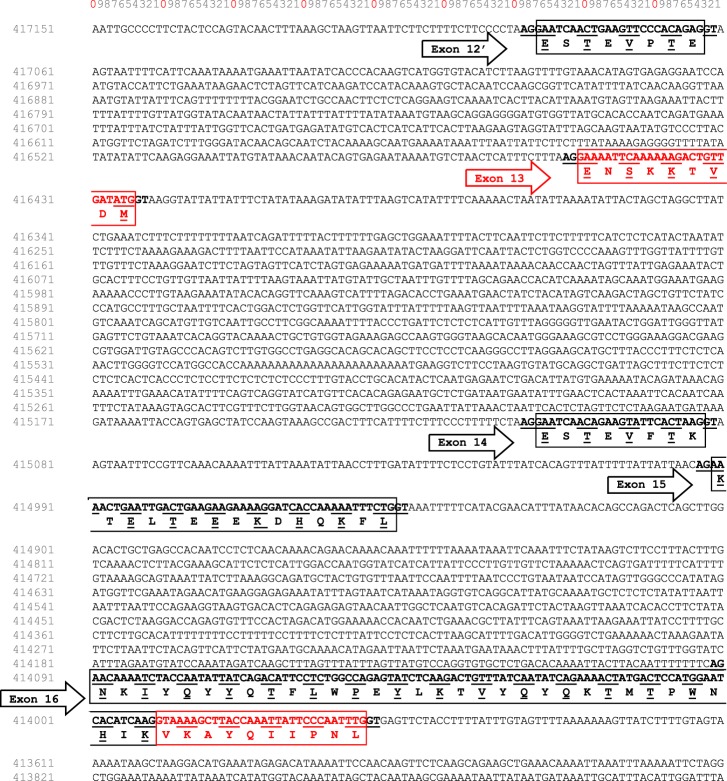
Nucleotide sequence view (from 417151 to 413731) of *C*. *dromedarius* (breed Arabia) taken from the unplaced genomic scaffold of CSN1S2 (LOC105090951). Already known exons 12′, 14, 15 and 16 are given in black, and additional exon 13 and extension of 30 additional nucleotides of exon 16 are in red. Exon subdivisions are boxed with amino acid sequences beneath. Intron donor and acceptor splice sites are underlined.

A deep and comprehensive analysis of the dromedary camel *CSN1S2* gene sequence available in GenBank (gi|742343530|ref|NW_011591251.1|), overlaying exon 12′ (ESTEVPTE) to exon 14 (ESTEVFTK) allowed identifying a 27-nucleotide sequence corresponding to exon 13 (Fig. 5). This sequence is flanked with consensus splice sites at the beginning (GTG/AAG) and end (polypyrimidine tract followed by XAG) of intron sequences. Therefore, this exon is included or not during the course of camel α_s2_-CN pre-mRNA processing. This is possibly due to the weakness (presence of purine in the polypyrimidine tract at the 3′-end of the upstream intron) of the acceptor splice sequence. The short transcript (without exon 13) encodes the 193 aa residues (including the signal peptide) described by Kappeler *et al*.^22^ and the long transcript (with exon 13) codes for UP1 (202 aa including signal peptide). The mature protein corresponding to UP1 is named thereafter α_s2_-CNsv1.

**Figure 6:**
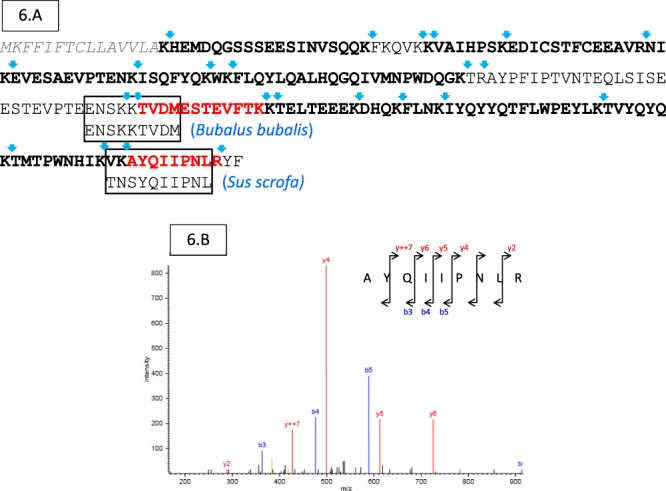
Identification and characterization of UP1 and UP2 as splicing variants of α_s2_-CN by LC-MS/MS analysis. (**A**) Camel α_s2_-CN full-length sequence is given and its coverage (81%) from peptides identified by LC-MS/MS analysis is in bold. Blue arrows indicate a cleavage of camel α_s2_-CN by trypsin. Tryptic peptides indicating the presence of exon 13 and extension of exon 16 are in red. Camel α_s2_-CN peptide sequences encoded by exon 13 and by the extension of exon 16 matching with *Bubalus bubalis* (UniProt KB accession number E9NZN2) and *Sus scrofa* (UniProt KB accession number P39036) are framed. The signal peptide is in italics and in grey. (**B**) Validation of the additional peptide sequence (AYQIIPNLR) with five and three ions from the “y” (including y7 double charged: y + + 7) and “b” series, respectively.

To confirm such an additional exon 13 hypothesis, detection of α_s2-_CN peptides after trypsin action was performed using liquid chromatography coupled to tandem mass spectrometry (LC-MS/MS). A tryptic peptide composed of 12 aa residues TVDMESTEVFTK (Fig. 6), identified through the *Bubalus bubalis* α_s2_-CN sequence (UniProt KB accession number E9NZN2), was attributed to two coherent arranged sequences (ENSKKTVDM and ESTEVFTK) encoded by exons 13 and 14, respectively. The sequence is identical to that of the *Bos taurus* (UniProt KB accession number P02663). The presence of a TVDM peptide sequence confirmed the existence of transcripts having included exon 13 during the course of pre-mRNA processing. Therefore, the existence of an exon 13 alternatively spliced in the camel *CSN1S2* gene was successfully confirmed both at the protein (LC-MS and LC-MS/MS) and at the nucleotide (cDNA sequencing and genome data) levels. The same cDNA sequences encoding α_s2_-CN with and without a 27-nucleotide additional sequence (exon 13) were found in all individual samples analyzed, including *C*. *bactrianus*, *C*. *dromedarius*, and hybrids.

Concerning the second unknown protein detected (UP2) that showed molecular masses comprised between 23,046 Da and 23,286 Da with n and n + 3 phosphate groups, in LC-ESI-MS, the mass difference observed was 1,140 Da, relative to the 8P-11P α_s2_-CN protein reported by Kappeler and co-workers^22^. LC-MS/MS analysis revealed the occurrence of a 9 aa-long peptide (AYQIIPNLR) matching with the C-terminal sequence of *Sus scrofa* α_s2_-CN (NP_001004030.1), strongly suggesting that mRNA described by Kappeler *et al*.^22^ was in fact the result of a cryptic splice site usage occurring in the antepenultimate exon of the camel *CSN1S2* gene (Fig. 6).

Examination of intron sequence downstream of exon 16 (Fig. 5) highlighted a 30-nucleotide segment: GTA AAA GCT TAC CAA ATT ATT CCC AAT TTG encoding 10 aa residues (VKAYQIIPNL). The intron donor splice site following the previously considered ending sequence of exon 16 CACATCAAG│GTAAA was recognized by the spliceosome machinery to generate the protein described by Kappeler *et al*.^22^. Alternatively, a second downstream intron donor splice site (CCC AAT TTG│GTGAG), which also fulfils all requirements of a splicing recognition signal, may also be used as well (Fig. 5). As a result, this alternative splicing event is responsible for the occurrence of two mature peptide chains, the first one made of 178 aa residues (21,906 Da with 8P), and the second one 10 aa residues longer (23,046 Da with 8P). The mature protein corresponding to UP2 is named thereafter α_s2_-CNsv2. Interestingly, the 10 aa residue peptide (VKAYQIIPNL) included in the C-terminal part of the camel protein due to this alternative splicing event was highly similar with the porcine (TNSYQIIPNL) and donkey (TNSYQIIPVL) α_s2_-CN sequences. Recently a shorter α_s2_-CN isoform, in which a deletion of the heptapeptide YQIIPVL, was reported in donkey milk^25,26^.

### Cross-species comparison of the gene encoding α_s2_-CN and primary transcript maturation

**Figure 7:**
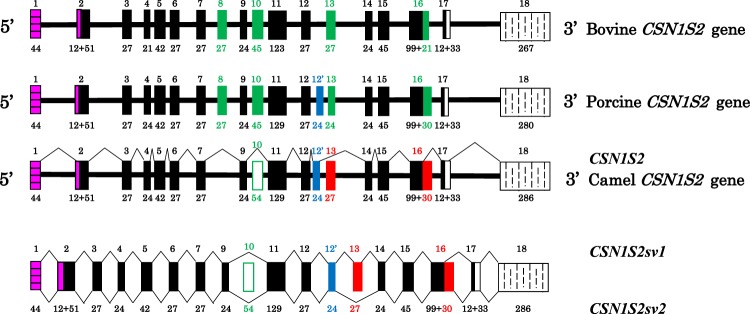
Structural organization of the bovine, porcine and camel *CSN1S2* transcription units and splicing patterns for camel (*CSN1S2*, *CSN1S2sv1* and *CSN1S2sv2*). *CSN1S2* corresponds to the splicing pattern characterized by Kappeler *et al*.^22^. Solid bars represent introns, and exons are depicted by blocks: 5′UTR and noncoding sequence are given in pink, leader peptide and coding frame are in black, exons absent from the camel protein are in green, exons absent from the bovine protein are in blue, exons found in our study are in red, and 3′UTR in white. Exons and exon sequences present in bovine and porcine *CSN1S2* but which were absent from the camel until now are highlighted in green, while exons present in the camel and pig are in blue. Exon 13 and the extension of exon 16 identified in this study are in red. Exon numbering (referring to bovine) and sizes (in bp) are indicated at the top, and at the bottom of the structures, respectively.

Comparative analysis of camel *CSN1S2* gene organization with orthologous bovine and pig genes is illustrated in Fig. 7. The first camel α_s2_-CN sequence published by Kappeler *et al*.^22^ lacks three peptide sequences encoded in cattle by exons 8 (EYSIGSSSE), 10 (EVKITVDDKHYQKAL), and 13 (ENSKKTVDM) composed of 27, 45 and 27 nucleotides, respectively. By contrast, exon 12′ that encodes in camel and lama a peptide of 8 aa residues (ESTEVPTE), was believed to be missing in the bovine counterpart, while it was present in the porcine genome, coding for the EPVSSSQE peptide. Surprisingly, we succeed in finding a putative exon 12′, encoding the octapeptide VSANSSQE, in intron 12 of the bovine gene. However, the downstream GTAAG donor splice site flanking this putative exon 12′ is mutated in GCAAG, apparently preventing its recognition as such as an exon. On the other hand, we failed to find a putative exon 8 in intron 7 of the camel gene. Exon 10 is present both in bovine and pig *CSN1S2* genes. In addition, it is also present in intron 9 of the camel gene, being 9 nucleotides longer than in the other species (Fig. 7), and bounded upstream and downstream by canonical intron consensus sequences. However, even though it seems to be perfectly eligible for splicing, we did not find any transcript nucleotide sequence, nor tryptic peptides at the protein level, signing its presence in multiple mRNA encoding α_s2_-CN. By contrast, as demonstrated in the present study, exon 13 was actually present in some camel *CSN1S2* transcripts, as well as the peptide sequence it is coding for in isoform α_s2_-CNsv1. Finally, the camel *CSN1S2* gene, just as its lama counterpart^23^, is made up of at least 17 exons, since we have no objective demonstration of the usage of exon 10, whereas its bovine and porcine counterparts are made up of 18 and 19 exons, respectively. Since a further exon sequence (exon 7′) occurs in the Equidaes *CSN1S2B* gene (not in *CSN1S2-*like *A*), we can hypothesize that the *CSN1S2* gene can comprise up to 20 exons with different combinatory splicing schemes across species. Interestingly, sequence alignments revealed that within the bovine intron 7, as well as in camels and pigs, the sequence corresponding to horse and donkey exon 7′ is partially deleted.

**Figure 8:**
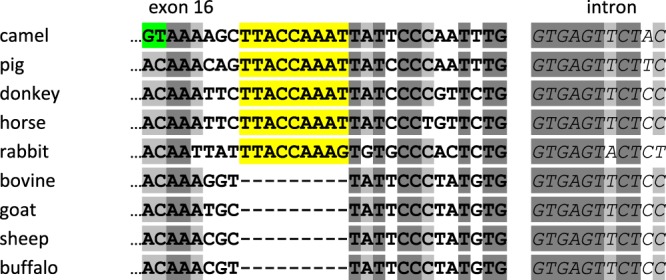
Alignment of nucleotide sequences of exon 16 3′-end and downstream intron across nine species. Accession numbers of different species are: camel (NCBI Gene ID: 105090951), pig (NCBI Gene ID: 445515), donkey (NCBI Gene ID: 106835119), horse (NCBI Gene ID: 100327035), rabbit (NCBI Gene ID: 100009288), bovine (NCBI Gene ID: 282209), goat (NCBI Gene ID: 100861229), sheep (NCBI Gene ID: 443383), and buffalo (NCBI Gene ID: 102395699). Exon sequences are in bold, intron sequences are in italics. Perfectly conserved nucleotides are dark-grey shaded. Nucleotides identical in more than eight animal species are light-grey shaded. Dashes in ruminants indicate missing nucleotides that are highlighted in yellow in the other species. The dinucleotide GT, highlighted in green in the camel sequence, generates the preferential site of splicing occurring within exon 16 that leads to the main α_s2_-CN isoform first described by Kappeler *et al*.^22^.

Genomic and mRNA analyses carried out previously demonstrated that deletions of aa residues in CN across species occurred essentially by exon skipping during the processing of the primary transcripts^7,8,23,27–29^. This event, leading to a shortening of the peptide chain length, is caused by weaknesses in the consensus sequences, either at the 5′ and/or 3′ splice junctions or at the branch point, or both^7^. Therefore, alternative splicing has to be regarded as a frequent event, mainly in α_s_-CN encoding genes, for which the coding region is divided into many short exons. Usage of cryptic splice sites is also responsible for the occurrence of multiple transcripts and finally for generating a protein molecular diversity. For example, the peptide sequence (VKAYQIIPNL) encoded by the “extension” of 30 nucleotides at the 3′ end of exon 16, not previously detected in camel nor in lama α_s2_-CN, was shown here to be alternatively included in camel *CSN1S2* transcripts. Extending the comparison to other species including ruminants, pigs and Equidaes, we show that the true donor splice site (GTGAG…) defining the end of exon 16 and common to the considered species (Fig. 8), is located 30 nt downstream of that preferentially used in Camelidaes. In other words, the isoform corresponding to UP2/α_s2_-CNsv2 is the genuine protein, whereas the isoform first described^22^ corresponds to the protein arising from the usage of a cryptic splice site internal to an exon.

The combination of both splicing events such as exon skipping and cryptic splice site usage generates more transcript isoforms in the same species and is responsible for the differences across species in the aa sequences of α_s2_-CN. However, regarding α_s2_-CN in camels we were not able to detect any transcript in which both exon 13 and the extension of exon 16 were present (α_s2_-CNsv3). That does not mean that this structure does not exist, even though the protein corresponding to both events was not detected in LC-MS profiling. Therefore, given that such an isoform is putatively present at a very low level, cloning PCR fragments and screening of a significant number of clones should probably make it possible to identify such a transcript.

### Phosphorylation level enhances camel α_s2_-CN isoform complexity

The non-phosphorylated peptide chain of the mature α_s2_-CN protein, which comprises 178 aa residues, yields a molecular weight of 21,266 Da^22^. Compared with other Ca-sensitive CNs, α_s2_-CN is the most phosphorylated with 12 potential phosphorylation sites and it is therefore likely to be the major transporter of Ca-phosphate.

Structural characterization of the α_s2_-CN fraction and relevant mRNA analyses has demonstrated that camel α_s2_-CN should be theoretically present in milk as a mixture of at least 18 isoforms derived from three mature peptide chains comprising 178 (α_s2_-CN), 187 (α_s2_-CNsv1, UP1) and 188 (α_s2_-CNsv2, UP2) aa residues originating from alternative splicing phenomena (Fig. 4). Each splicing variant should display six phosphorylation levels ranging between 7 and 12P groups. Based on LC-ESI-MS data, we identified 14 phosphorylation isoforms. Surprisingly, even though an additional peptide sequence does not provide further phosphorylation sites, the predominant phosphorylation level of each peptide isoform was not the same: 8P for α_s2_-CN, 8P for α_s2_-CNsv1, and 10P for α_s2_-CNsv2. The addition of 10 aa residues in the C-terminal part of α_s2_-CNsv2 might induce conformational changes in the protein facilitating the modification of definite phosphorylable sites. Multiple non-allelic variants produced from at least three different mRNA were shown to occur in all thirty Kazakh individuals analyzed, apparently indicating a stabilized mechanism for the production of protein isoforms of different lengths, structures and possibly biological activities.

With 11 potentially phosphorylated aa residues matching the S/T-X-A motif, camel α_s2_-CN displays the highest phosphorylation level, as mentioned by Ryskaliyeva *et al*.^8^. To reach such a phosphorylation level, besides the nine SerP, two putative Threonine residues (T118 and T132) should be phosphorylated. However, in all the Kazakh milk samples analyzed in LC-ESI-MS we found α_s2_-CN with up to 12P groups. This means that at least another S/T residue that does not match the canonical sequence recognized by the mammary kinase(s), is potentially phosphorylated. According to Allende *et al*.^30^ the sequence S/T-X-X-A is in agreement with the minimum requirements for phosphorylation by the CN-kinase II (CK2). In this regard, it is critical to highlight that the A residue in this site, usually E or D, can be replaced by SerP or ThrP. Two T residues, namely T39 and T129 in the camel α_s2_-CN fully meet the requirements of the above-mentioned motif and might be phosphorylated. Such an event is the only possible hypothesis to reach 12P for camel α_s2_-CN. Since these two kinases are very likely secreted, the idea that phosphorylation at T39/T129 may occur in the extracellular environment cannot be excluded. This warrants further investigation. Fam20C, which is very likely the major secretory pathway protein kinase^31^, might be responsible for the phosphorylation of S and T residues within the S/T-X-A motif, whereas a CK2-type kinase might be responsible for phosphorylation of the T residue within an S/T-X-X-A motif. This was in agreement with the hypothesis put forward by Bijl *et al*.^32^ and Fang *et al*.^33^, who suggest, from phenotypic correlations and hierarchical clustering, the existence of at least two regulatory systems for phosphorylation of α_s_-CN. Interestingly, twelve phosphorylation sites were also predicted in llama α_s2_-CN^23^, including two Threonine residues at T118 (instead of T114 as erroneously mentioned) and T141 (also T141 in camel α_s2_-CNsv1). Phosphorylation sites matching the S/T-X-A motif in llama α_s2_-CN are actually 12. Indeed, S122 (llama’s numbering) that has been predicted as phosphorylated^23^ does not meet the criteria required by the S/T-X-A consensus motif and cannot be phosphorylated. By contrast, T128, which is substituted by a methionine residue (M) in the camel α_s2_-CNsv1, is potentially phosphorylated provided S130 has been phosphorylated before. On the contrary, sites potentially phosphorylated by a second kinase (CK2-type) identified in the camel sequence are also present in the llama sequence and therefore the phosphorylation level that could be reached in this species is potentially13P.

### Alternate splicing isoforms of camel α_s2_-CN increase its ability to generate potential bioactive peptides

A growing number of genes encoding milk proteins displays complex patterns of splicing, thus increasing their coding capacity to generate an extreme protein isoform diversity from a single gene. It is well established that milk proteins represent a reservoir of biologically active peptides^13,34,35^, capable of modulating different functions. Therefore, beside genetic polymorphisms, the molecular diversity generated by differential splicing mechanisms can increase its content.

**Figure 9:**
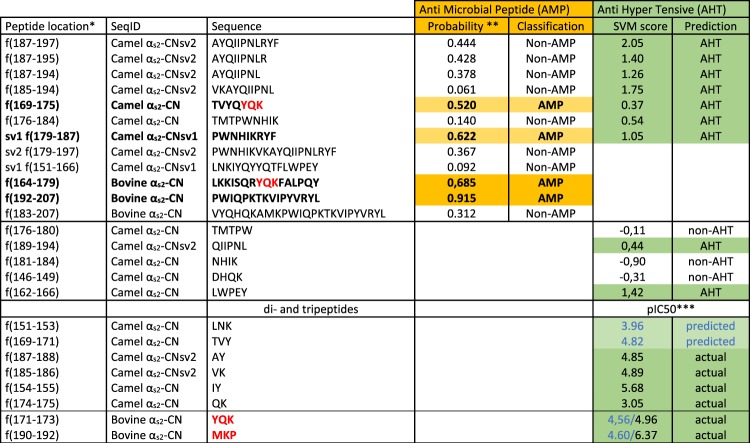
*In silico* analyses of α_s2_-CN peptides for antimicrobial (yellow) and antihypertensive (green) activities. *Peptide location is given in the longest camel amino acid sequence (putative a_*s2*_*-CNsv3)*. **Probability > 0.5 = *Predicted AMP*. ***pIC50 = *−logIC50 with IC50* = *peptide concentration (μmol/L) necessary to inhibit the angiotensin converting enzyme (ACE) activity by 50%*. *SVM (support vector machine) score: threshold* = *0*^17^. *Tripeptides YQK and MKP recently identified as an antihypertensive peptide*^43,52^ *are bolded and in red*.

To evaluate this possibility, we undertook to search for bioactive peptides encrypted in the different camel α_s2_-CN isoforms, using an *in silico* approach. Since alternative splicing events impact the C-terminal part of the molecule (f(150–197)) which seems, in addition, to be the most accessible domain of the bovine protein^15,36^, we therefore focused our attention on this region. Previous studies performed on the bovine α_s2_-CN have demonstrated that this casein is the least accessible in the micelles and that a limited number of tryptic peptides were released from its C-terminal part^37,38^; of which some were subsequently shown to display antibacterial properties^15^. The first antibacterial peptide isolated from bovine α_s2_-CN (f(150–188) of the mature protein), inhibiting the growth of *Escherichia coli* and *Staphylococcus carnosus*, was called casocidin-I^39^. Two distinct antibacterial domains f(164–179) and f(183–207), also located in the C-terminal part of the molecule, were subsequently isolated from a peptic hydrolysate of bovine α_s2_-CN^40^. It is worth noting that in our prediction analyses (Fig. 9), the bovine peptide f(164–179) displays a rather high probability (0.685) to have an antimicrobial (AMP) activity; whereas peptide f(183–207) for which a probability of 0.312 was found, would not have such an activity. In contrast, peptide f(192–207) is by far the one with the highest probability (0.915) to exhibit an AMP activity.

The picture is less positive with regard to the corresponding camel sequences, since peptides f(179–197) and f(179–187), according to the splicing variant (α_s2_-CN sv2 and α_s2_-CN sv1, respectively), as well as f(151–166) from α_s2_-CN sv1, compared with the bovine α_s2_-CN f(164–179), gave more contrasted results (Fig. 9). Given the magnitude of the splicing events occurring in the camel α_s2_-CN pre-mRNA, it is not surprising that it would impact biological properties of α_s2_-CN C-terminal peptides, including antimicrobial activity, since several aa residues of this region were shown to be essential regarding AMP activity^39,40^. Indeed, the importance of specific amino acids (P and R residues) at the C-terminus of the bovine milk-derived α_s2_-CN f(183–207) peptide for its antibacterial activity against the food-borne pathogens *Listeria monocytogenes* and *Cronbacter sakazakii*, was recently demonstrated^41^. Nevertheless, this *in silico* screening remains a predictive approach, aimed at identifying sequences that would be potentially bioactive. It is therefore necessary to confirm experimentally, and possible discordances may occur between *in silico* and *in vitro* results. It is not because the sequence of a peptide is predicted as potentially bioactive that it will be actually active *in vitro* and if it is active *in vitro*, this does not mean that even though it will be active *in vivo*. McCann *et al*.^42^ identified 5 peptides from chymosin digests of a bovine sodium caseinate, all being once again from the C-terminal end of α_s2_-CN, including f(164–207), f(175–207) and f(181–207), and showing *in vitro* antibacterial activity against *Listeria innocua*. However, they stressed that it was not excluded that these cationic peptides may lose their antibacterial activity *in vivo*. From all these studies it appears, nevertheless, that the C-terminal part of α_s2_-CN was predicted to yield peptides with defensin-like activity, which may aid the immune system in fighting bacteria^15^.

Interestingly, further bioactive peptides with different properties such as AHT (Anti Hyper Tensive) activity were identified from camel α_s2_-CN (Fig. 9). Indeed, according to the splicing patterns, including or not exon 16 extension, two peptide sequences (KTMTPWNHIKRYF and KTMTPWNHIKVKAYQIIPNLRYF) occur within the C-terminal part of the molecule (Fig. 4), thus giving rise to different peptides after digestion by proteolytic enzymes from the digestive tract, including pepsin, trypsin and chymotrypsin (Supplementary Data S2). Several peptides, related to the inserted VKAYQIIPNL decapeptide characterizing camel α_s2_-CNsv2, were *in silico* identified as AHT peptides involved in the angiotensin I-converting enzyme (ACE) inhibitory activity, with SVM (Support Vector Machine) scores >1 (Fig. 9). Two ACE-inhibitory dipeptides (f(185–186): VK and f(187–188): AY) were found exclusively in camel α_s2_-CNsv2 (and in the putative camel α_s2_-CNsv3). Interestingly, the AY dipeptide was also found in the B variant of the camel α_s1_-CN^21^. A novel ACE inhibitory peptide (YQK) exhibiting an IC_50_ of 11.1 µM was recently isolated from a pepsin and trypsin hydrolysate of bovine α_s2_-CN^43^. An oral administration, using a rodent hypertensive model, revealed a significant decrease of systolic blood pressure, thus demonstrating its AHT effects. Such a tripeptide sequence also occurs in the C-terminal part of the camel α_s2_-CN.

To summarize, the data reported here allowed identifying UP1 and UP2 detected in our previous study^1^ as splicing isoforms of α_s2_-CN (α_s2_-CNsv1 and α_s2_-CNsv2, respectively). These isoforms arise from different processing of the *CSN1S2* primary transcript, giving rise to the insertion of exon 13 in α_s2_-CNsv1 and a downstream extension of exon 16 in α_s2_-CNsv2. Thus, α_s2_-CN was shown to be a mixture of at least 16 isoforms differing in polypeptide chain length and phosphorylation levels, identified in both *Camelus* species (*C*. *bactrianus* and *C*. *dromedarius*), as well in hybrids. Such a situation is not specific to Camelids and is frequently observed in most of the mammalian species, particularly in small ruminants and Equidae. Little is known about the mechanisms identifying alternatively spliced exons. Do those deletions/insertions in camel α_s2_-CN simply reflect the lack of accuracy of an intricate processing mechanism whenever mutations induce conformational modifications of pre-mRNA, preventing the normal progress of the splicing process? There are more and more evidences to support the hypothesis that *cis*-acting sequences, both in introns and exons, are involved in the control of this process.

Despite the extreme conservation of the organization of the “casein” locus during the course of evolution (Fig. 1), the sequences of the proteins encoded by each of the genes that compose this locus have rapidly evolved. Given the exon modular structure of messenger RNAs, the real similarity between α_s2_-CN across species is significantly higher than it appears at first whether the exon modular structure is taken into account (Fig. 4). The apparent divergence is in fact largely due to a splicing combinatorial assembly of exons specific of each species, as previously suggested by Martin *et al*.^44^, as far as α_s1_-CN is concerned. Therefore, differential splicing, as well as genetic polymorphisms as described with camel α_s1_-CN^21^, generate a molecular diversity of sequences increasing the ability of camel caseins to generate potentially bioactive encrypted peptides.

## Methods

### Ethics Statements

All animal studies were carried out in compliance with European Community regulations on animal experimentation (European Communities Council Directive 86/609/EEC) and with the authorization of the Kazakh Ministry of Agriculture. Milk sampling was supervised by a veterinarian accredited by the French Ethics National Committee for Experimentation on Living Animals.

### Milk Sample Collection and Preparation

Raw milk samples were collected during morning milking on healthy dairy camels belonging to two species: *C*. *bactrianus* (n = 72) and *C*. *dromedarius* (n = 65), and hybrids (n = 42) at different lactation stages, ranging between 30 and 90 days postpartum. Camels grazed on four various natural pastures from different regions of Kazakhstan, namely Almaty (AL), Shymkent (SH), Kyzylorda (KZ), and Atyrau (ZKO). Whole-milk samples were centrifuged at 3,000 *g* for 30 min at 4°C (Allegra X-15R, Beckman Coulter, France) to separate fat from skimmed milk. Samples were quickly frozen and stored at −80 °C (fat) and −20 °C (skimmed milk) until analysis.

### Selection of Milk Samples

Thirty milk samples: *C*. *bactrianus* (n = 10), *C*. *dromedarius* (n = 10), and hybrids (n = 10)) were selected for LC-ESI-MS analysis from the 179 camel milks collected in a previous study^1^, based on lactation stages and number of parities (from 2 to 14). The most representative eight milk samples (*C*. *bactrianus*, n = 3, *C*. *dromedarius*, n = 3, and hybrids, n = 2) were analyzed by LC-MS/MS (LTQ-Orbitrap Discovery, Thermo Fisher Scientific) after a tryptic digestion of bands, excised from each track, between 20 and 30 kDa of SDS-PAGE.

### RNA Extraction from Milk Fat Globules

Total RNA was extracted from MFG using TRIzol*®* and TRIzol® LS solutions (Invitrogen, Life Technologies), respectively, according to the original manufacturer’s protocol modified as described by Brenaut *et al*.^45^.

### First-Strand cDNA Synthesis and PCR Amplification

First-strand cDNA was synthesized from 5 to 10 ng of total RNA primed with oligo(dT)20 and random primers (3:1, vol/vol) using Superscript III reverse transcriptase (Invitrogen Life Technologies Inc., Carlsbad, CA) as described previously^1^. Primer pairs, purchased from Eurofins (Eurofins genomics, Germany), were designed using published *Camelus* nucleic acid sequences (NCBI, NM_001303566.1 for α_s1_-CN and NM_001303561.1 for α_s2_-CN). The forward primers for α_s1_-CN and α_s2_-CN amplification were 5′-CTTACCTGCCTTGTGGCTGT-3′ (starting from nucleotide 61, located in exon 2 of α_s1_-CN mRNA) and 5′-TCATTTTTACCTGCCTTTTGGCTGT-3′ (starting from nucleotide 71, located in exon 2 of α_s2_-CN mRNA), respectively. The reverse primers were 5′-GTGGAGGAGAAATTTAGAGCAT-3′ (terminating at nucleotide 751 of α_s1_-CN mRNA located in the last exon) and 5′-CGATTTTCCAGTTGAGCCATA-3′ (terminating at nucleotide 692 of α_s2_-CN mRNA located in the last exon), respectively. Thus, the amplified fragments cover regions of 691 nucleotides for α_s1_-CN and 622 nucleotides for α_s2_-CN, including the sequence coding the mature proteins, with genomic reference to the published sequences (NCBI, NM_001303566.1 for α_s1_-CN and NM_001303561.1 for α_s2_-CN). Five (two *C*. *bactrianus*, one *C*. *dromedarius*, and two hybrids) samples representative of the 30 camel milks analyzed in LC-MS, were selected for amplification of α_s1_-CN and α_s2_-CN cDNA by RT-PCR and sequencing. Amplicons were sequenced from both strands with primers used for PCR according to the Sanger method by Eurofins (Eurofins genomics, Germany).

### Identification of proteins and validation of peptides by LC-MS/MS Analysis

In order to identify the different α_s1_-CN and α_s2_-CN isoforms, mono dimensional electrophoresis (1D SDS-PAGE), followed by trypsin digestion and LC-MS/MS analysis, was used. After a long migration (10 cm) in 1D SDS-PAGE, bands (1.5 mm^3^) migrating in the range of 20–30 kDa, were cut on each of the eight gel lanes, and analyzed as described by Henry *et al*.^46^ and Saadaoui *et al*.^47^.

### LC-ESI-MS

Fractionation of camel milk proteins and determination of their molecular masses were performed by coupling RP-HPLC to ESI-MS (micrOTOF^TM^ II focus ESI-TOF mass spectrometer; Bruker Daltonics). Twenty μL of skimmed milk samples were clarified by addition of 230 μL of clarification solution 0.1 M bis-Tris buffer pH 8.0, containing 8 M urea, 1.3% trisodium citrate, and 0.3% DTT. Clarified milk samples (25 μL) were directly injected onto a Biodiscovery C5 reverse phase column (300 Å pore size, 3 µm, 150 × 2.1 mm; Supelco, France) and analyzed as described by Miranda *et al*.^48^.

### *In silico* release of Peptides using PeptideCutter and BIOPEP analyses

Protein sequences of α_s2_-CN from *Bos taurus* (entry P02663), *Lama glama* (entry A0A0D6DR01) and *Camelus dromedarius* (entry O97944 and new sequences identified in the present study) were selected from the Protein Knowledge Base (UniProtKB, ExPASy Bioinformatics Resource Portal) available at www.uniprot.org. Each sequence was then subjected to *in silico* release of peptides by pepsin (pH 1.3), pepsin + trypsin and pepsin + trypsin + chymotrypsin using “PeptideCutter”, a resource available at www.expasy.org. Thereafter, each α_s2_-CN sequence was entered in the “PeptideCutter”. After cutting the sequences, a list of probable peptides with cleavage sites, length and amino acid sequence of peptides was established. BIOPEP analyses were then performed at https://omictools.com/biopep-tool by selecting the available option “Peptide Prediction Software Tools”. Peptide Structure Prediction/AHTpin^17^ and Antimicrobial Peptide Prediction/Antimicrobial Peptide Scanner^49^ (AMP Scanner Vr.2) sections were used one by one for prediction of the peptides with the sought properties.

## Supplementary information

S1 and S2
