# Supplementary material for: Alternative splicing events expand molecular diversity of camel CSN1S2 increasing its ability to generate potentially bioactive peptides
Source: Sci Rep. 2019 Mar 27;9:5243. doi: 10.1038/s41598-019-41649-5 (PMC6437144; doi:10.1038/s41598-019-41649-5)
Supplement: Supplementary file 1 — S1 and S2 [file 41598_2019_41649_MOESM1_ESM.pdf]

Alternative splicing events expand molecular diversity of camel CSN1S2 increasing its ability to generate potentially bioactive peptides

Ryskaliyeva A, Henry C, Miranda G, Faye B, Konuspayeva G and Martin P

Supplementary figure 1 (S1)

Bioanalyser profile of camel CSN1S2 PCR products

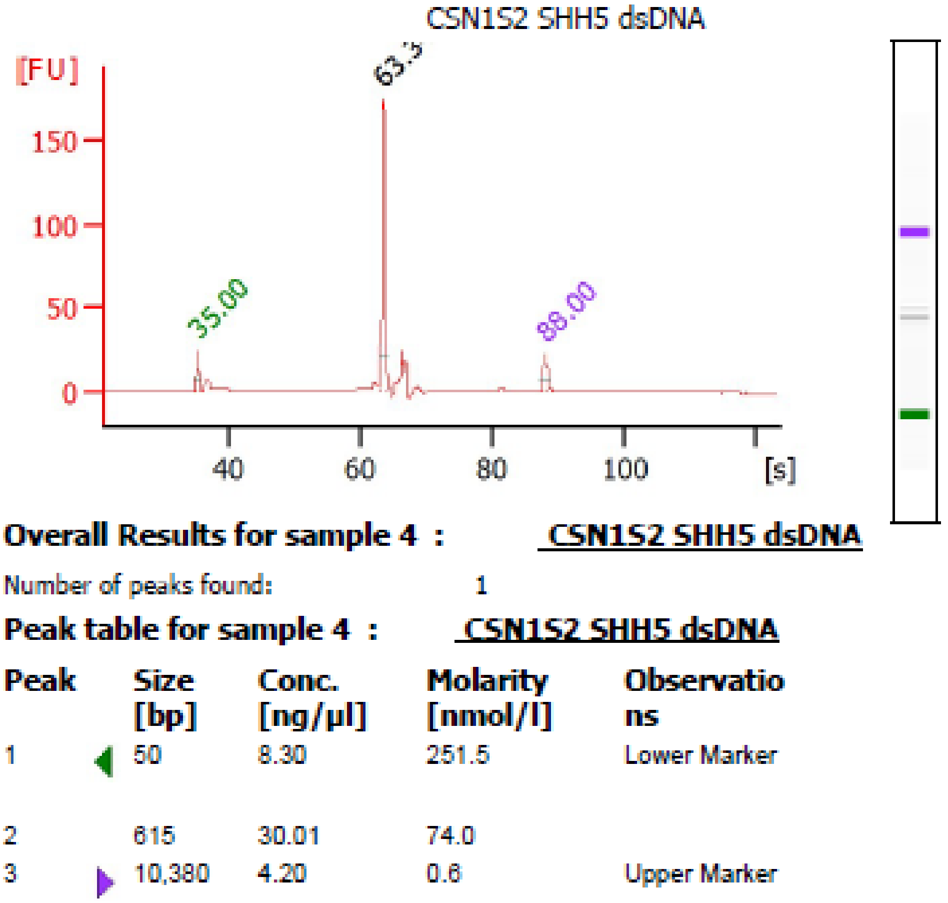

Overall Results for sample 4 : CSN1S2 SHH5 dsDNA

Number of peaks found: 1

Peak table for sample 4 : CSN1S2 SHH5 dsDNA

Peak		Size [bp]	Conc. [ng/µl]	Molarity [nmol/l]	Observations
1	◀	50	8.30	251.5	Lower Marker
2		615	30.01	74.0	
3	▶	10,380	4.20	0.6	Upper Marker

Supplementary figure 2 (S2)

Digestion profile of the longest camel CSN1S2 (putative sv3, 197 aa), including exon 13 and downstream intron extension of exon 16, by Pepsin (pH1.3) +Chymotrypsin-high specificity (C-term to [FYW], not before P) + Trypsin

```
                              Tryps
                            Tryps|
                         Tryps  ||
                  Ch hi Pn1.3|  ||   Ch hi Pn1.3    Tryps
    Tryps       Pn1.3 Tryps||   ||  Tryps Pn1.3| Tryps  |

        |                  |||  ||      |     ||     |  |
        KHEMDQGSSSEESINVSQQKFKQVKKVAIHPSKEDICSTFCEEAVRNIKEVESAEVPTEN
    1   ---------+---------+---------+---------+---------+---------+   60


                     Pn1.3
                    Pn1.3|
                  Pn1.3 ||
           Ch hi Pn1.3| ||
        Ch hi Pn1.3  || ||
             Tryps|  || ||
            Ch hi||  || ||
           Tryps|||  || ||                Ch hi
         Ch hi ||||  || ||               Pn1.3|
  Ch hi Pn1.3| ||||  || ||             Tryps  ||
       Pn1.3|| ||||  || ||           Tryps |  ||    Pn1.3
    Tryps  ||| ||||  || ||     Ch hi   | |  ||    Pn1.3|

        |  ||| ||||  || ||         |   | |  ||       ||
        KISQFYQKWKFLQYLQALHQGQIVMNPWDQGKTRAYPFIPTVNTEQLSISEESTEVPTEE
   61   ---------+---------+---------+---------+---------+---------+   120


                                                               Ch hi
                                                          Tryps    |
                                                        Ch hi |    |
                                                      Ch hi | |    |
                                                   Tryps  | | |    |
                                                  Pn1.3|  | | |    |
                                           Ch hi Pn1.3||  | | |    |
                                      Ch hi Pn1.3    |||  | | |    |
                                            Pn1.3|   |||  | | |    |
                                         Ch hi  ||   |||  | | |    |
                                        Ch hi|  ||   |||  | | |    |
                                      Ch hi ||  ||   |||  | | |    |
                                    Tryps | ||  ||   |||  | | |    |
                           Ch hi Pn1.3  | | ||  ||   |||  | | |    |
                                Tryps|  | | ||  ||   |||  | | |    |
                            Tryps   ||  | | ||  ||   |||  | | |    |
                       Pn1.3    |   ||  | | ||  ||   |||  | | |    |
                    Tryps  |    |   ||  | | ||  ||   |||  | | |    |
                   Tryps|  |    |   ||  | | ||  ||   |||  | | |    |
           Ch hi Pn1.3 ||  |    |   ||  | | ||  ||   |||  | | |    |
       Tryps         | ||  |    |   ||  | | ||  ||   |||  | | |    |
      Tryps|    Pn1.3| ||  |    |   ||  | | ||  ||   |||  | | |    |

          ||        || ||  |    |   ||  | | ||  ||   |||  | | |    |
        NSKKTVDMESTEVFTKKTELTEEEKDHQKFLNKIYQYYQTFLWPEYLKTVYQYQKTMTPW
  121   ---------+---------+---------+---------+---------+---------+   180


             Ch hi Pn1.3
           Ch hi       |
         Tryps |  Tryps|
       Tryps | | Pn1.3||

           | | |     |||
        NHIKVKAYQIIPNLRYF
  181   ---------+-------   197
```

Digestion profile of the camel CSN1S2 sv1 (187 aa), including exon 13, by Pepsin (pH1.3) +Chymotrypsin-high specificity (C-term to [FYW], not before P) + Trypsin

```
                           Tryps
                          Tryps|
                       Tryps  ||
                Ch hi Pn1.3|  ||   Ch hi Pn1.3    Tryps
  Tryps        Pn1.3 Tryps||  ||  Tryps Pn1.3| Tryps  |

      |                  |||  ||      |     ||     |  |
      KHEMDQGSSSEESINVSQQKFKQVKKVAIHPSKEDICSTFCEEAVRNIKEVESAEVPTEN
  1   ---------+---------+---------+---------+---------+---------+   60
```

```
                   Pn1.3
                  Pn1.3|
                Pn1.3 ||
         Ch hi Pn1.3| ||
      Ch hi Pn1.3  || ||
           Tryps|  || ||
          Ch hi||  || ||
         Tryps|||  || ||               Ch hi
       Ch hi ||||  || ||              Pn1.3|
Ch hi Pn1.3| ||||  || ||           Tryps  ||
     Pn1.3|| ||||  || ||         Tryps |  ||    Pn1.3
  Tryps  ||| ||||  || ||     Ch_hi   | |  ||   Pn1.3|

      |  ||| ||||  || ||         |   | |  ||       ||
      KISQFYQKWKFLQYLQALHQGQIVMNPWDQGKTRAYPFIPTVNTEQLSISEESTEVPTEE
 61   ---------+---------+---------+---------+---------+---------+   120
```

```
                                                             Ch hi
                                                        Tryps    |
                                                      Ch hi |    |
                                                    Ch_hi | |    |
                                                 Tryps  | | |    |
                                                Pn1.3|  | | |    |
                                         Ch hi Pn1.3||  | | |    |
                                    Ch hi Pn1.3    |||  | | |    |
                                         Pn1.3|    |||  | | |    |
                                       Ch hi ||    |||  | | |    |
                                      Ch hi| ||    |||  | | |    |
                                    Ch hi || ||    |||  | | |    |
                                  Tryps | || ||    |||  | | |    |
                         Ch hi Pn1.3  | | || ||    |||  | | |    |
                              Tryps|  | | || ||    |||  | | |    |
                          Tryps   ||  | | || ||    |||  | | |    |
                     Pn1.3    |   ||  | | || ||    |||  | | |    |
                  Tryps  |    |   ||  | | || ||    |||  | | |    |
                 Tryps|  |    |   ||  | | || ||    |||  | | |    |
         Ch hi Pn1.3 ||  |    |   ||  | | || ||    |||  | | |    |
     Tryps         | ||  |    |   ||  | | || ||    |||  | | |    |
    Tryps|    Pn1.3| ||  |    |   ||  | | || ||    |||  | | |    |

        ||        || ||  |    |   ||  | | || ||    |||  | | |    |
      NSKKTVDMESTEVFTKKTELTEEEKDHQKFLNKIYQYYQTFLWPEYLKTVYQYQKTMTPW
121   ---------+---------+---------+---------+---------+---------+   120
```

```
       Ch hi
      Tryps|
     Tryps||

         |||
      NHIKRYF
181   -------   187
```

Digestion profile of the major camel CSN1S2 isoform (178 aa, first described by Kappeler et al., 1998) by Pepsin (pH1.3) +Chymotrypsin-high specificity (C-term to [FYW], not before P) + Trypsin

```
                           Tryps
                          Tryps|
                       Tryps  ||
                Ch hi Pn1.3|  ||   Ch hi Pn1.3    Tryps
  Tryps        Pn1.3 Tryps||  ||  Tryps Pn1.3| Tryps  |

      |                  |||  ||      |     ||     |  |
      KHEMDQGSSSEESINVSQQKFKQVKKVAIHPSKEDICSTFCEEAVRNIKEVESAEVPTEN
  1   ---------+---------+---------+---------+---------+---------+   60
```

```
                   Pn1.3
                  Pn1.3|
                Pn1.3 ||
         Ch hi Pn1.3| ||
      Ch hi Pn1.3  || ||
           Tryps|  || ||
          Ch hi||  || ||
         Tryps|||  || ||               Ch hi
       Ch hi ||||  || ||              Pn1.3|
Ch hi Pn1.3| ||||  || ||           Tryps  ||
     Pn1.3|| ||||  || ||         Tryps |  ||    Pn1.3
  Tryps  ||| ||||  || ||     Ch hi   | |  ||   Pn1.3|

      |  ||| ||||  || ||         |   | |  ||       ||
      KISQFYQKWKFLQYLQALHQGQIVMNPWDQGKTRAYPFIPTVNTEQLSISEESTEVPTEE
 61   ---------+---------+---------+---------+---------+---------+   120
```

```
                                                          Ch hi
                                                         Tryps|
                                                        Tryps||
                                                    Ch hi   |||
                                               Tryps    |   |||
                                             Ch hi |    |   |||
                                           Ch hi | |    |   |||
                                        Tryps  | | |    |   |||
                                       Pn1.3|  | | |    |   |||
                                Ch hi Pn1.3||  | | |    |   |||
                           Ch hi Pn1.3    |||  | | |    |   |||
                                Pn1.3|    |||  | | |    |   |||
                              Ch hi ||    |||  | | |    |   |||
                             Ch hi| ||    |||  | | |    |   |||
                           Ch hi || ||    |||  | | |    |   |||
                         Tryps | || ||    |||  | | |    |   |||
                Ch hi Pn1.3  | | || ||    |||  | | |    |   |||
                     Tryps|  | | || ||    |||  | | |    |   |||
                 Tryps    ||  | | || ||    |||  | | |    |   |||
            Pn1.3    |   ||  | | || ||    |||  | | |    |   |||
         Tryps  |    |   ||  | | || ||    |||  | | |    |   |||
        Tryps|  |    |   ||  | | || ||    |||  | | |    |   |||
Ch hi Pn1.3 ||  |    |   ||  | | || ||    |||  | | |    |   |||
     Pn1.3| ||  |    |   ||  | | || ||    |||  | | |    |   |||

         || ||  |    |   ||  | | || ||    |||  | | |    |   |||
      STEVFTKKTELTEEEKDHQKFLNKIYQYYQTFLWPEYLKTVYQYQKTMTPWNHIKRYF
121   ---------+---------+---------+---------+---------+--------   178
```
